# Phosphorus Removal from Dirty Farmyard Water by Activated Anaerobic-Digestion-Derived Biochar

**DOI:** 10.1021/acs.iecr.2c02668

**Published:** 2022-12-05

**Authors:** Chen Zhang, Shuzhuang Sun, Shaojun Xu, Chris Johnston, Chunfei Wu

**Affiliations:** † School of Chemistry and Chemical Engineering, Queens University Belfast, BelfastBT7 1NN, United Kingdom; ‡ School of Chemistry, Cardiff University, CardiffCF10 3AT, United Kingdom; § UK Catalysis Hub, Research Complex at Harwell, DidcotOX11 0FA, United Kingdom; ∥ The Agri-Food and Biosciences Institute, BelfastBT9 5PX, United Kingdom

## Abstract

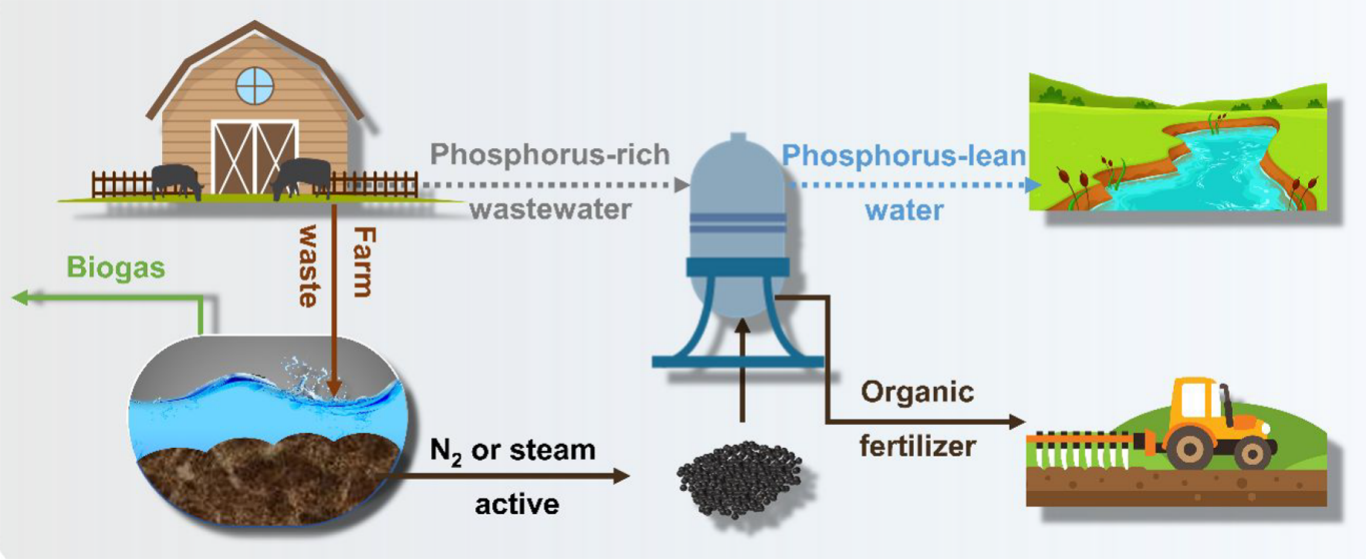

The management of anaerobic digestate is important to realize the value of the waste and enhance the whole system sustainability of anaerobic digestion. In this study, the phosphorus treatment of dirty irrigation water by biochar samples derived from digestate of anaerobic digestion were investigated. The biochars were further activated by steam activation with different duration time and KOH activation with different introducing ratios; the textural properties of biochars were optimized after activation from the aspect of biochar characterization. Notably, AD-N_2_ demonstrates a remarkable adsorption effect of phosphorus, with an adsorption efficiency of 8.99 mg g^–1^. Besides the effect of biochar dosage on phosphorus removal, adsorption kinetics and thermodynamic isotherms are studied. According to the adsorption kinetics, the adsorption of phosphorus from dirty water fits the Elovich equation (*R*^2^ = 0.95). Furthermore, the thermodynamic isotherm results illustrate the process of phosphorus removal by biochar is endothermic (Δ*H*^0^ = 17.93 kJ mol^–1^) and spontaneous (Δ*S* = 96.24 J mol^–1^ K^–1^). Therefore, this work suggests a promising solution to phosphorus-related environmental challenges in industry and agriculture.

## 1 Introduction

Phosphorus-rich water can cause severe environmental problems.^1^ The increasing phosphorus concentration in surface and groundwater systems can cause eutrophication due to rapid socio-economic development.^2^ Consequently, phosphorus pollution in the ecological system can gradually evolve into the phenomenon of eutrophication, which could have devastating impacts on aquatic ecosystems^3^ and environmental sustainability.^4^ Typically, the main origins of phosphorus entering the environment are related to industrial discharge^5^ (phosphorus-contained detergents), agricultural activities^6^ (waste from animal husbandry and misuse of phosphorus fertilizers), and municipal sewage. Hence, it is essential to avoid phosphorus discharge and remove phosphorus from the eutrophic aqueous system.

Nowadays, the typical phosphorus removal and adsorption methods are chemical precipitation,^7^ biological methods,^8^ and solid sorbents adsorption.^9^ Solid sorbent adsorption is the comprehensive method to achieve phosphorus removal and due to the wide variety of adsorbents. The leading candidates of the adsorbents are fly ash,^10^ steel slag,^11^ activated carbons,^12^ and metal oxides.^13^ However, although metal oxides (especially iron oxides) are recognized as state-of-the-art adsorbents for phosphorus removal, the high cost and the difficulties of sorbent recycling are the main problems.^14^ Therefore, compared to metal oxide sorbents, carbonous materials are promising for phosphorus adsorption due to their low costs, environmentally benign, and easy modification.

Biochar, a carbonous material, has been used as an efficient solid sorbent for phosphorus removal, carbon capture and soil amendment.^15^ It is a carbon-rich, highly aromatic, and stable solid substance produced from biomass pyrolysis under total or partial hypoxia.^16^ Nonactivated biochar has lower phosphorus removal abilities than activated biochar,^17^ which is attributed to the negative surface charge that promotes electrostatic repulsive interaction with phosphorus molecules.^18^ Consequently, the biochar activation process suggests a positive effect on phosphorus adsorption performance via modifying the surface area, functional group, and porosity of biochar sorbents.^19^ Moreover, the phosphorus adsorption performance of biochar is also related to the feedstock of biochars. According to the reported works,^20^ nonactivated biochar has various phosphorus removal abilities in a range between 1.37 and 193 mg g^–1^.^20^ However, not all biochars reduce phosphorus in wastewater, and some biochars with high P content would discharge phosphorus from the inside when immersed in an aqueous solution.^21^ Therefore, suitable biomass feedstock and biochar activation are essential for phosphorus removal.

Digestate from anaerobic digestion (AD) is a common waste produced by biogas plants and agricultural activities,^22^ and the management methods of the digestate are mainly as a fertilizer and a soil amendment compound.^23^ However, combustion and landfill of AD digestate may create various environmental concerns potentially,^24^ such as greenhouse gas emissions and eutrophication of soil and water systems, due to the poor biological stability and nutrient loss. Hence, to explore alternative applications of AD digestate in various fields, AD digestate has been applied to generate biochar by hydrothermal carbonization^25^ and one-pot synthesis.^23^ Presently, AD digestate biochar has been utilized in the field of fertilizer,^26^ carbon capture,^23^ and fuel production.^27^ To our knowledge, AD biochar has the advantages of large production yield, abundant elements, easy preparation, and luxuriant surface functional groups, which are the potential possibilities for applying AD biochar in wastewater treatment. However, although Alberto et al.^28^ reported that digestate-generated biochar could be determined as a potential sorbent for phosphorus adsorption,^28^ the deficiency of evidence about phosphorus removal by AD digestate biochar still exists.

Notably, though the adsorption of phosphorus from aqueous solution by biochar has been investigated over the past decades,^20,29^ studying the P removal performance of biochars when dealing with actual wastewater is rare.^30^ Herein, the phosphorus solution in this work was obtained from real agricultural irrigation dirty water. Meanwhile, to promote the idea of using wastes to treat wastes, AD digestate biochar was chosen to explore its feasibility in phosphorus removal from dirty water. Therefore, the present study has examined the phosphorus removal by AD digestate biochar under different sorbent activation conditions, considering the effect of steam activation and chemical activation on phosphorus adsorption from dirty water.

## 2 Experimental Section

### 2.1 Activation of the Biochar

The AD digestate solids were derived from AFBI by anaerobically digesting dairy cow slurry and silage. The digestate was dehydrated with the use of a FAN Press Screw Separator. The crude AD digestate solids were ground and sieved, then dried to a powder in an oven overnight at 105 °C. The dry precursor was named AD-Origin. Around 5 g of AD-Origin was placed into a quartz tube, and a syringe pump was used to introduce water at a flow rate of 10 mL h^–1^ to introduce water vapor carried by nitrogen gas at a gas flow rate of 100 mL min^–1^. The horizontal tube furnace was then heated to 550 °C before steam was introduced into the reactor. The activation processes were conducted at 550 °C for 30 and 60 min, respectively, and the corresponding activated samples were named AD-Steam-0.5h and AD-Steam-1.0h. Additionally, the sample not treated with water vapor is regarded as AD-N_2_.

Dried AD digestate solids were mixed with KOH powder by mortar and pestle at the ratio of 1:0.5 and 1:1. Afterward, the sample was calcined at 550 °C for 1 h in the horizontal tube furnace under an atmosphere of N_2_ (100 mL min^–1^). Then, the sample was washed with hydrochloride solution and deionized water until neutral and dried at 110 °C overnight. The final samples were denoted as AD-KOH-1:0.5 and AD-KOH-1:1.

### 2.2 Measurement of Phosphorus in Dirty Water

Typically, definite AD biochar was immersed into the sewage supplied by Agri-Food and Biosciences Institute (AFBI), stirring the mixture solution at a particular time. First, 0.1 g samples of different biochars are immersed in 20 mL of dirty water to determine the phosphorus removal ability of various biochar samples. Next, we added different amounts of AD-N_2_ and AD-Steam-1.0h separately in 20 mL of dirty water to evaluate the effect of biochar dosages on P removal ability and percentage. Moreover, 0.25 g of biochar was added to 100 mL of dirty water to study the kinetics and thermodynamics (adsorption temperatures are 298, 308, and 318 K) of phosphorus removal by AD biochars.

From the perspective of phosphorus detection, a small amount of solution was collected for each sample at different adsorption times. 0.2 mL sample solutions were added for specific sample vials with 2.8 mL of distilled water, and 3 mL phosphorus determination reagent (the preparation of the regent is shown in the Supporting Information) was added to the vials. The samples were kept in a 45 °C water bath for 25 min to ensure the samples entirely achieve coloration. The phosphorus coloration with determination reagent has a characterized peak at 820 nm detected by UV–vis spectrometer chromatography (PerkinElmer Lambda 800). The phosphorus concentration can be calculated using a calibration curve and UV–vis adsorption values (Figure S1).

### 2.3 Characterization

The content and composition of C, H, and N in the biochar samples were determined by CHNS Element Analyzer (PerkinElmer PE2400). Temperature-programmed oxidation (TPO) analysis was performed using a TA Instruments TGA 2950 thermogravimetric analyzer (TGA) under an air atmosphere to measure moisture, ash, and volatile matter contents. Around 20 mg of sample was heated to 850 °C at 10 °C min^–1^ under N_2_ flowing (100 mL min^–1^) to obtain the content of moisture and volatiles. The presence of functional and aromatic groups on the surface of biochar samples was determined by ATR-FTIR (Agilent Cary 630 spectrometer), and the spectra in the range of 3000–1400 cm^–1^ band were analyzed. The multipoint BET surface area was measured with a Quantachrome instrument, using the adsorption of N_2_ at 77 K. The surface morphology of the material was determined by scanning electron microscope (SEM) using JEOL JSM-6610LV, and the distribution of elements on the surface of samples was monitored using an energy-dispersive X-ray analyzer (SEM-EDX).

## 3 Results and Discussion

### 3.1 Biochar Characterization

C, H, O, N, and metallic elements are the main components of biochar.^31^Table 1 provides an overview of the elementary constituents of AD biochar. The precursor has 41.46% carbon element, and H can reach 5.45%, which has hydrogen content similar to the other digestate.^23,32^ Additionally, proximate analysis results detected by TG and TPO are shown in Table 1, which indicates that the volatile matters of different types of biochar decreased significantly, from 79.71% to about 60% after pyrolysis. After pyrolysis and activation, biochar samples exhibit higher C content values than AD-Origin because volatile matter escapes under moderate pyrolysis temperature. Besides, AD-KOH-1:1 has 33.24% carbon content, possibly due to the high quantity of KOH introduced during activation, and the potassium content transformed to metal oxide as ash content. Even though the hydrochloride solution was used to extract potassium from biochar, it is difficult to entirely eliminate the potassium element in KOH-acctivated biochar sample.

**Table 1:** Proximate Analysis Results and CHNS Contents (wt %)

	proximate analysis				element analysis				
sample name	moisture (%)	volatile matter (%)	fixed carbon (%)	ash (%)	C	H	N	S	*S*_BET_(m^2^ g^–1^)^a^
AD-Origin	4.75	33.21	46.50	15.54	41.46	5.45	1.85	<0.30	3.13
AD-N_2_	4.14	6.13	54.52	35.21	52.44	1.36	1.58	<0.30	7.78
AD-Steam-0.5h	2.29	5.16	54.79	37.76	49.61	1.38	1.41	<0.30	20.52
AD-Steam-1.0h	3.80	4.98	60.37	30.85	54.19	1.68	1.41	<0.30	117.94
AD-KOH-1:0.5	4.28	5.93	53.27	36.52	43.29	1.55	1.22	<0.30	39.52
AD-KOH-1:1	1.74	6.44	47.24	44.58	33.24	1.62	0.86	<0.30	189.48

a BET surface area.

The BET surface area of the AD biochar sample is shown in Table 1. The dry precursor does not exhibit a promising surface area (3.13 m^2^ g^–1^), and the activated biochar samples show significant enhancement. Specifically, the surface area of physically activated biochar increased with the activation time postponed to 1.0 h, which suggests 117.94 m^2^ g^–1^. While the potassium hydroxide activated biochar shows the same trend of surface area change, it has a surface area of 189.48 m^2^ g^–1^ when the ratio of biochar and KOH is 1:1.

**Figure 1:**
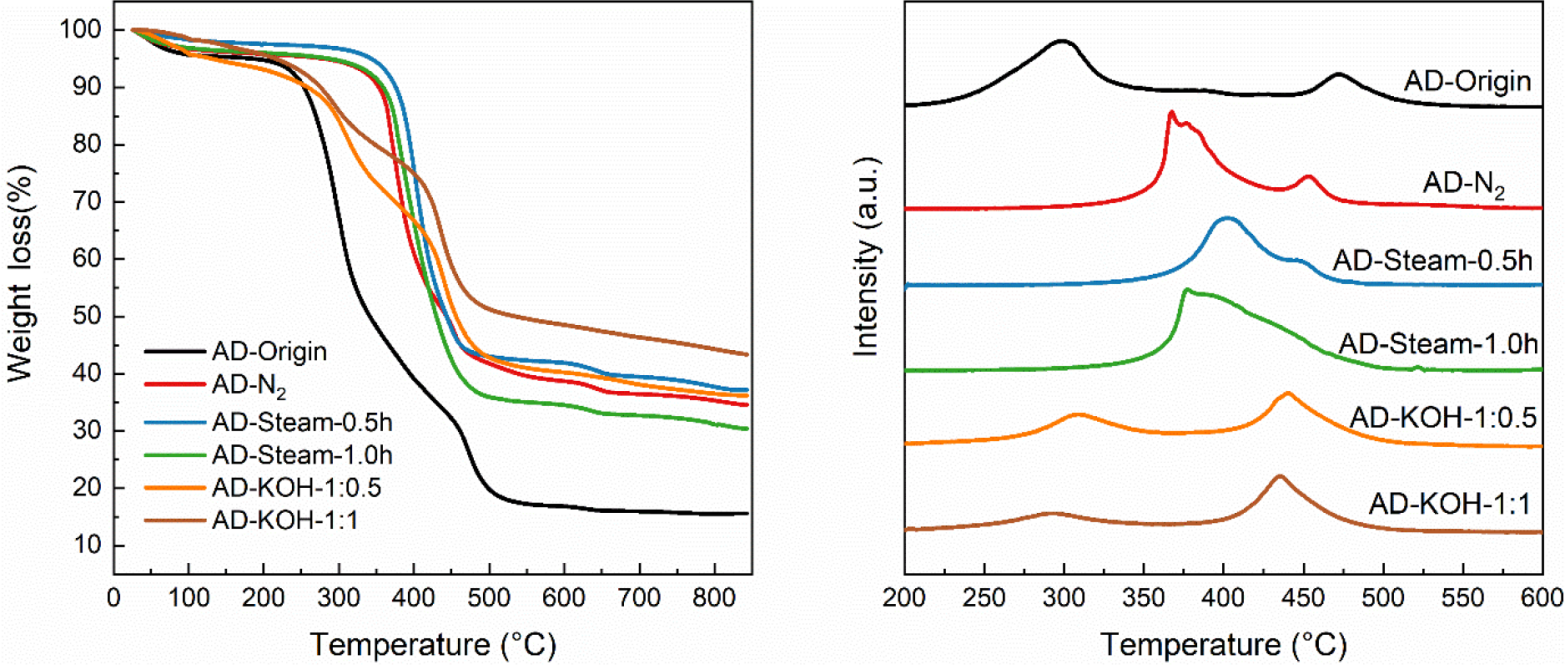
TGA-TPO and DTG-TPO profiles of the AD biochars with the varied activated method.

Furthermore, Figure 1 presents the results of TG and TPO analysis. The DTG-TPO profile of AD-Origin shows the oxidation peaks at 310 and 475 °C. However, the oxidation peaks are merged and shifted when AD-Origin is carbonized and activated. AD biochars generated under nitrogen and moisture show broad peaks in the range of 350–475 °C, while the oxidation peaks of KOH-activated biochar have a similar oxidation temperature range compared to AD-Origin. Generally, the lower temperature oxidation peak suggests the presence of volatile matter and amorphous carbon, and the peak at higher temperatures illustrates the oxidation of the main carbon of the biochar samples. The high oxidation temperature of biochar also suggests a more stable char structure,^33^ possibly because the cellulose and lignin within AD-Origin were oxidized during biochar production and activation.^34^

**Figure 2:**
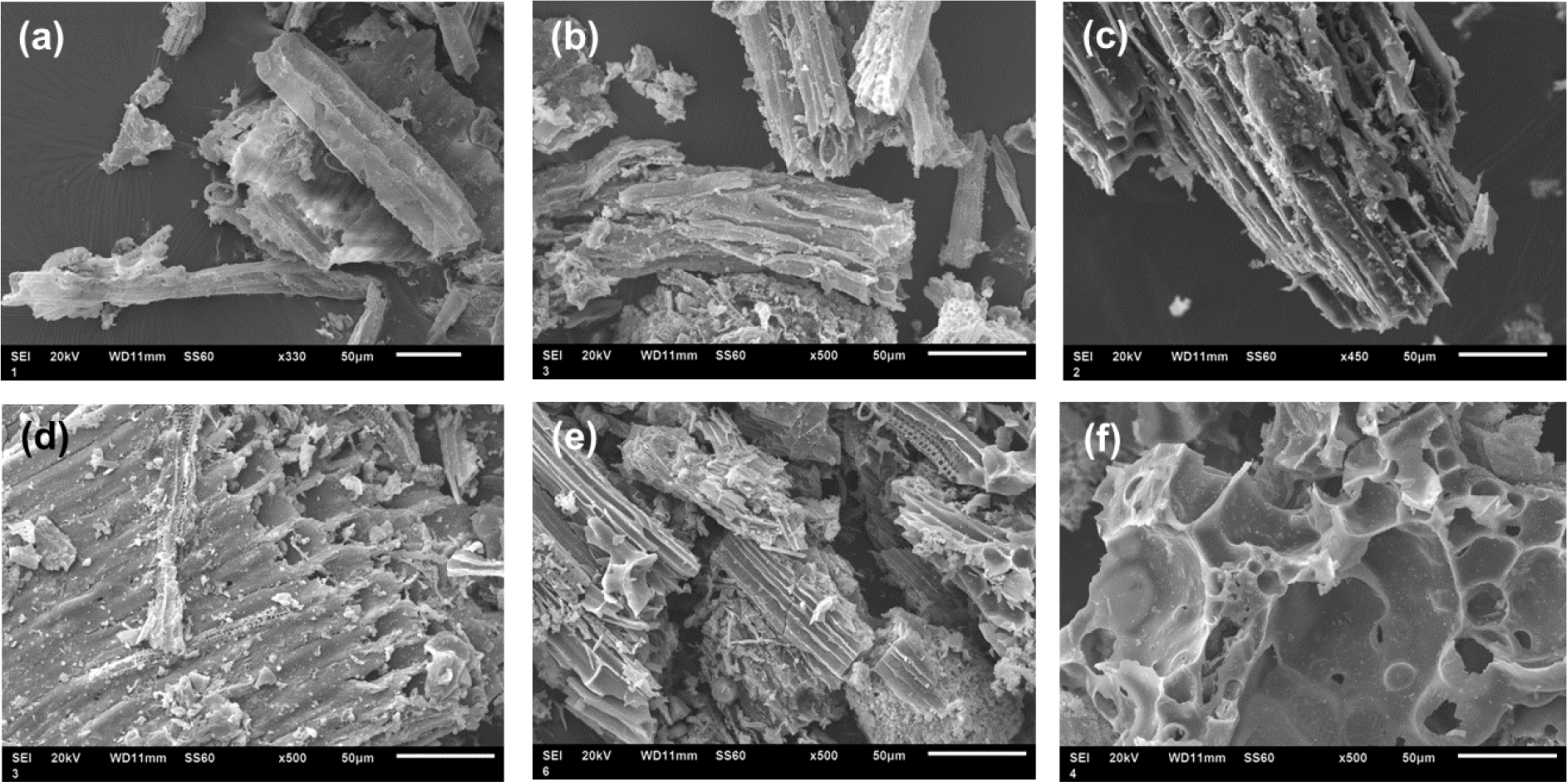
SEM micrographs of (a) AD-Origin, (b) AD-N_2_, (c) AD-Steam-0.5h, (d) AD-Steam-1.0h, (e) AD-KOH-1:0.5, and (f) AD-KOH-1:1.

Scanning electron microscopy (SEM) indicates the microcosmic morphology of AD biochars. Figure 2a shows the structure of AD-Origin, and the noncorrosive and less-porous form is revealed. Additionally, the tubular structure appeared on high-temperature pyrolysis biochar (AD-N_2_). Moreover, Figure 2c,d and 2e,f show intense contrast due to the effects of different activation methods on biochar morphological structure. Steam activation and KOH activation also formed tubular structures. More crucially, the canal structures facilitated the diffusion of KOH into the interior of biochar to generate porosity (Figure 2f).^35^

Furthermore, the distribution of elements in the biochar samples was analyzed by SEM-EDX, and the results are shown in Figure S2. The subtle color differences of C, N, and O demonstrate that the activation showed little effect on main element content changing. On the contrary, the content of the potassium element is dramatically elevated in AD-KOH-1:0.5 and AD-KOH-1:1 due to the introduction of the KOH.

**Figure 3:**
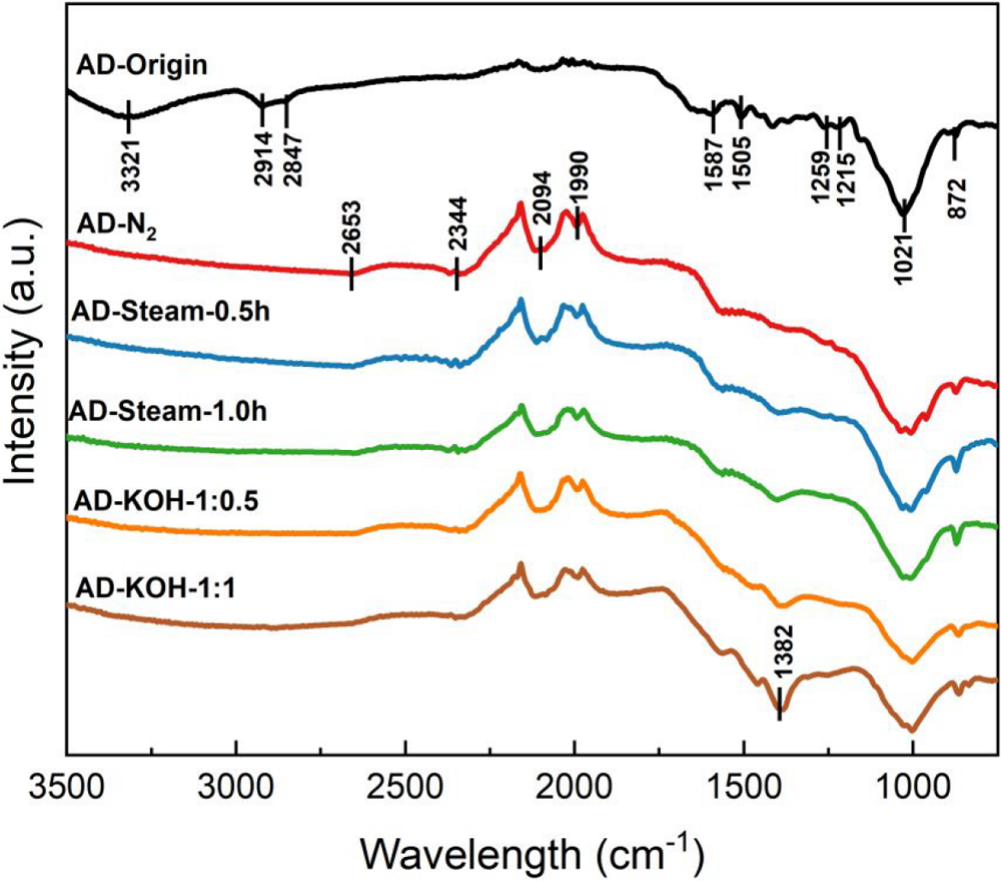
ATR-FTIR spectra of AD biochar samples.

Furthermore, FTIR is applied to understand the surface functional groups, including −OH, C=C, C–C, −CH_*x*_ (*x* = 1, 2, 3), C=O, and aromatic bonds. As shown in Figure 3, the FTIR results of AD-Origin illustrate the N–O stretching at 1587 and 1505 cm^–1^, while 1400–1000 cm^–1^ generally suggests oxygen-contained bending and the 1259 and 1215 cm^–1^ correspond to the C–O stretching of aromatic ester and alkyl aryl ether. Additionally, the wide strong appearance at 1021 cm^–1^ shows the CO-O–CO stretching of anhydride. Moreover, all the AD biochars exhibit a peak at 872 cm^–1^, which illustrates the N–H stretching.^23^ In addition, the activated AD biochars show a decrease of absorbance at a high adsorption wavelength range, whereas peaks in 2500–1900 cm^–1^ become the main characters of the sorbents. C=O groups have a specific peaks appearing in the ranges of 2000–1650 cm^–1^ and 1500–850 cm^–1^, which still contain the weak signal of aromatic C–H bending.^36^ It is worth noting that the central double bond groups, such as O=C=O, C=N, and C=C=C, give an intense appearance at 2400–2000 cm^–1^. Herein, the −OH stretching (3500–3200 cm^–1^) vanishing occurs when the AD biochars are activated. He et al.^37^ examined the appearance trend of −OH stretching and aliphatic −CH (2314 cm^–1^) and reported that the intensity of signals was reduced after increasing the pyrolysis temperature. Hence, the hydroxide surface group in AD biochar could easily be decomposed at 550 °C.

### 3.2 Phosphorus Adsorption

**Figure 4:**
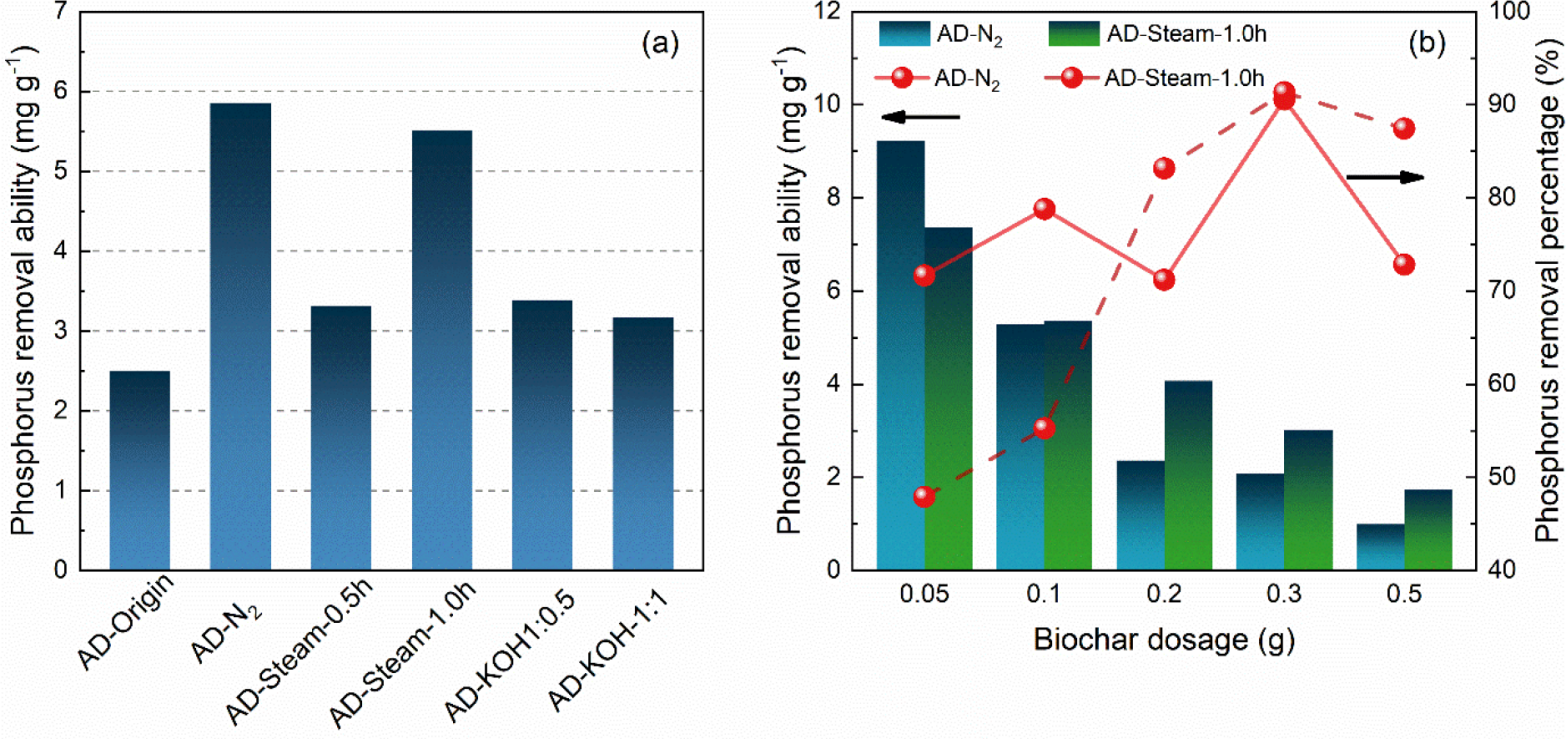
(a) Phosphorus removal ability of different activated AD biochar samples. (b) Effect of biochar dosage on phosphorus adsorption ability and percentage.

Figure 4a indicates phosphorus removal efficiency using 0.1 g of steam- and chemical-activated samples when 20 mL of dirty water was used for the adsorption at room temperature. AD-Origin, as the feedstock, demonstrates the ability to remove phosphorus in dirty water; however, the removal capacity is only 2.50 mg g^–1^. The removal abilities of the biochars are higher than AD-Origin. In particular, AD-N_2_ and AD-Steam-1.0h show high P removal capacities at 5.89 and 5.56 mg g^–1^, respectively. Generally, the physical-activated biochar samples (N_2_ and steam) are more active in removing phosphorus from the dirty water than KOH-activated biochar. AD-KOH-1:0.5 and AD-KOH-1:1 only show P removal abilities of 3.38 and 3.17 mg g^–1^, respectively. Typically, phosphorus uptake by biochar can not be attributed to intraparticle diffusion by surface textural structure.^38^ Although many studies have reported high phosphorus uptake due to the high surface area and well-developed mesoporous,^39^ the key contribution of P uptake should be ascribed to the electrostatic interaction between sorbent and phosphorus.^18^ Notably, mesopores (>3 nm) of activated carbon show a high correlation with the process of phosphorus adsorption in low P concentration.^40^ Therefore, KOH-activated biochars are unsuitable for adsorbing phosphorus because of the preference for micropores generation after KOH activation.^41^

As AD-N_2_ and AD-Steam-1.0h show excellent performance of P removal, these biochar samples were used to detect the effect of dosage on P removal ability. From Figure 4b, the phosphorus removal rates of the two selected biochar samples are significantly reduced when the biochar dosage is increased. The P removal capacity of AD-N_2_ decreases from 9.22 to 0.99 mg g^–1^. Moreover, for AD-Steam-1.0h, the ability of P removal is decreased from 7.37 to 1.73 mg g^–1^ when the amount of biochar increases from 0.05 to 0.5 g due to the enhanced adsorption sites.^42^ Furthermore, phosphorus removal percentage is evaluated as the typical standard to measure the performance of adsorbents in phosphorus adsorption, which is also enhanced with the augment of biochar dosage. AD-N_2_ phosphorus removal percentage is maintained at 72–91%; by contrast, the removal rate of AD-Steam-1.0h continues to increase from 47% to ∼90%, also ascribed to the increase of adsorption.^43^ Therefore, according to the removal efficiency and the sample input amount, the biochar dosage of 2.5 g L^–1^ was selected for the following kinetic and thermodynamic analysis.

It is noted that kinetics is important for phosphorus adsorption using biochar. In order to investigate the adsorption kinetics of phosphorus removal by biochar, the Elovich equation, pseudo-first-order, and pseudo-second-order models are used to describe the adsorption process. The Elovich equation is generally used for the adsorption of solid adsorbents and is typically uniquely representative of the absorption and release of phosphorus in the soil.^44^ Meanwhile, pseudo-first-order and pseudo-second-order kinetics models^45^ are widely modeled in adsorption experiments. Therefore, these three equations simulate the investigation of AD biochar adsorption of phosphorus in dirty water. For the parameters of the kinetic models, *c* (mg g^–1^) is the phosphorus removal ability (mg g^–1^); *c*_1_ (mg g^–1^) and *c*_2_ (mg g^–1^) are the theoretical efficiencies when adsorption reaches equilibrium; *a* and *b* are the constants for the Elovich equation; and *k*_1_ (g mg^–1^ min^–1^) and *k*_2_ (g mg^–1^ min^–1^) are the pseudokinetic equation constants.Elovich:
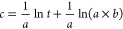
1Pseudo-first-order equation:
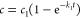
2Pseudo-second-order equation:
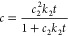
3

**Figure 5:**
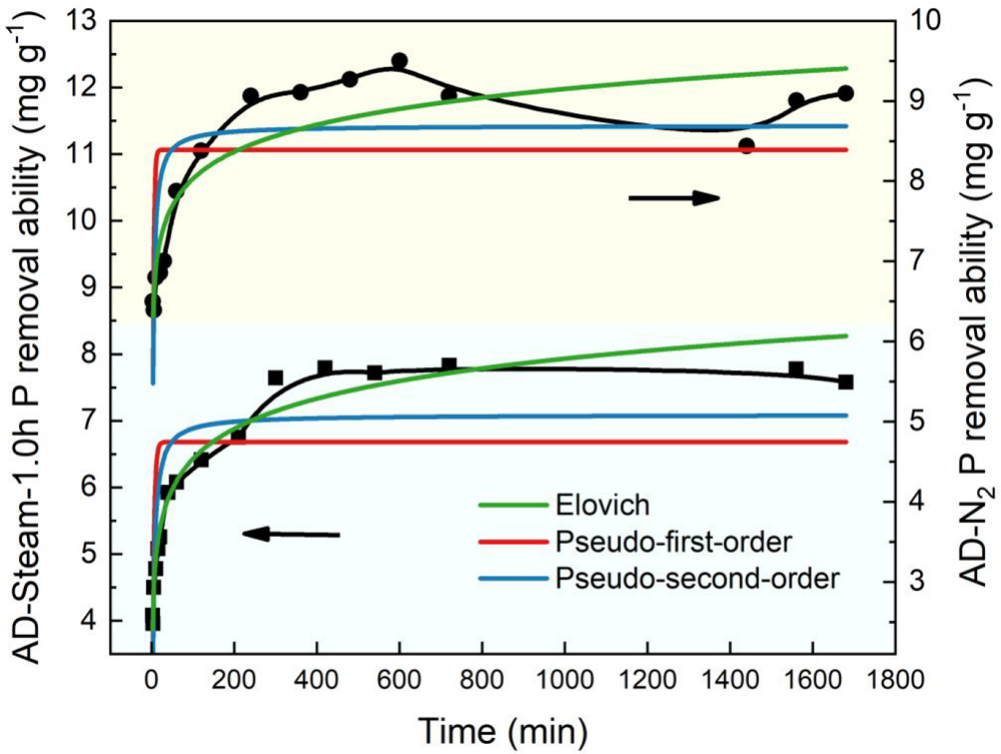
Adsorption kinetic fitting curves of phosphorus removal by AD-N_2_ and AD-Steam-1.0h.

As shown in Figure 5 and Table 2, AD-N_2_ and AD-Steam-1.0h reach stable phosphorus adsorption with increased stirring time. The adsorption processes are in equilibrium after ∼300 min, and the average adsorption abilities of AD-N_2_ and AD-Steam-1.0h are 8.99 and 7.98 mg g^–1^, respectively. Moreover, the theoretical P adsorption abilities of AD-N_2_ obtained from the pseudo-first-order and pseudo-second-order are 8.44 and 8.69 mg g^–1^, while the removal efficiencies of AD-Steam-1.0h shown from the pseudokinetic models are 6.68 and 7.09 mg g^–1^, respectively. Typically, the higher coefficient of determination (*R*^2^) of the pseudo-second-order model illustrates the preference of chemisorption processes during the phosphorus adsorption by biochar as in previous studies,^39,46^ which formed the valency forces entailed from exchanging electrons between sorbents and phosphorus.^47^ Besides the pseudokinetic models, the Elovich model shows a high correlation. The *R*^2^ value of the P adsorption process by AD-N_2_ is higher than 0.95, proving that the Elovich equation can simulate the adsorption process of phosphorus in dirty water by AD biochar sample. As reported by Wang,^42^ the Elovich kinetic is more suitable for the actual nonuniform surface with solid absorbent varied dramatically during the reaction process.

**Table 2:** Parameters of Kinetic Equations for ADB Phosphorus Removal

	Elovich			pseudo-first-order			pseudo-second-order		
	*a*	*b*	*R*^2^	*c*_1_	*k*_1_	*R*^2^	*c*_2_	*k*_2_	*R*^2^
AD-N_2_	1.56	140.63	0.95	8.44	0.36	0.34	8.69	0.06	0.64
AD-Steam-1.0h	1.53	122.98	0.96	6.68	0.26	0.49	7.09	0.05	0.75

Adsorption isotherms can be implemented to explore the adsorption capacity of AD biochars at various P concentrations under constant temperatures. Langmuir, Freundlich, and Langmuir–Freundlich are used for curve fitting. In the simulated equations, *c*_0_ (mg g^–1^) is the theoretical P removal ability; *K*_L_ (L mg^–1^), *K*_F_ (L mg^–1^), and *n* are constants for the Langmuir and Freundlich equations.Langmuir:
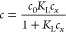
4Freundlich:
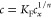
5Langmuir–Freundlich:
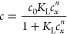
6

**Figure 6:**
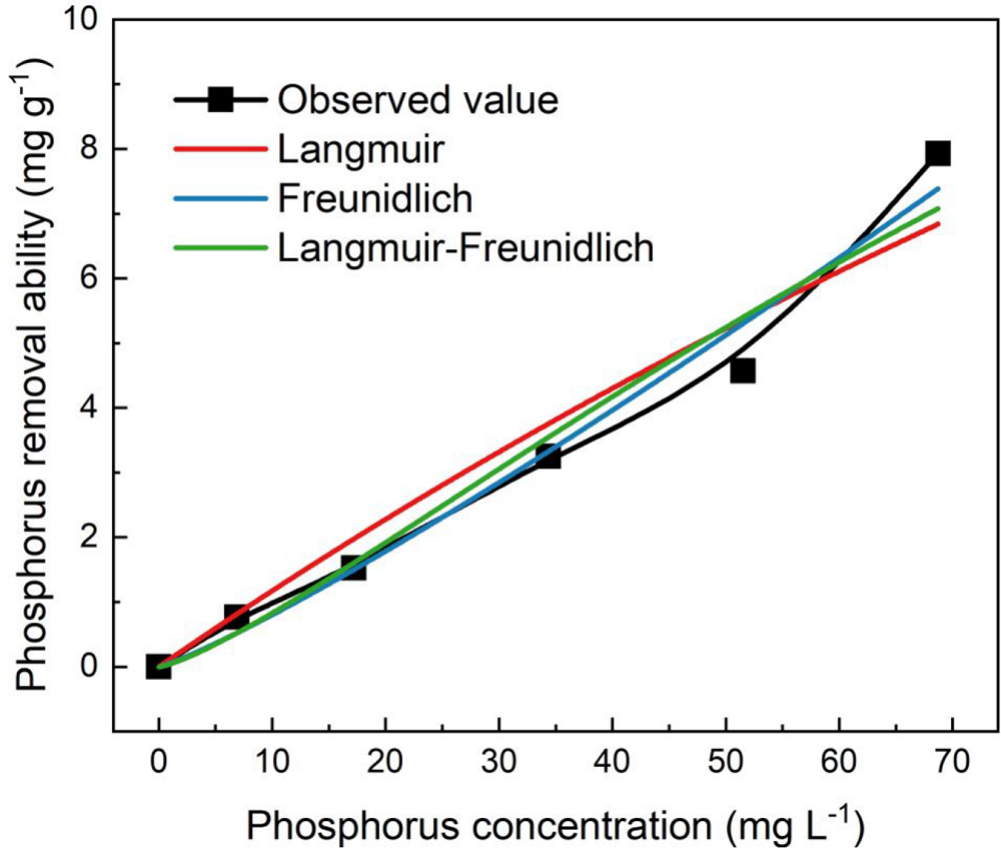
Adsorption isotherm and simulation of AD-Steam-1.0h for phosphorus removal at 298 K.

As shown in Figure 6, the phosphorus removal efficiency of AD-Steam-1.0h increases as the concentration of phosphorus increases. The results show that the theoretical maximum adsorption capacities obtained by Langmuir and Langmuir–Freundlich equations are 37.37 and 22.40 mg g^–1^ (shown in Table 3), with the *R*^2^ values of uptake higher than 0.95, respectively. The difference between the two theoretical adsorption abilities (*c*_0_) from Langmuir and Langmuir–Freundlich equations could not be neglected. The Langmuir model is generally suitable for homogeneous surface adsorption, and on the contrary, the Freundlich model is ideal for irregular morphology adsorbents (e.g., biochar).^48^ Typically, the Langmuir and Freundlich models are widely used to fit isotherms in phosphorus removal, so the results obtained from the Langmuir–Freundlich equation may be more reliable. However, although more observed data samples can obtain more accurate fitting results, the phosphorus concentration in the dirty water can not be concentrated. Hence, only five observed values are fitted for the isothermal models, and it is suggested that all three models (*R*^2^ > 0.9) are suitable to simulate phosphorus adsorption when the phosphorus concentration is in the range of 0–70 mg g^–1^.

**Table 3:** Parameters of Isotherm Adsorption Equations for AD-Steam-1.0h Phosphorus Removal

	*c*_0_	*K*_L_	*K*_F_	*n*	*R*^2^
Langmuir	37.37	0.0034			0.949
Freundlich			0.078	0.925	0.953
Langmuir–Freundlich	22.40	0.0022		1.278	0.966

The Gibbs free energy was used to detect the adsorption thermodynamics of AD-Steam-1.0h phosphorus removal at 298, 308, and 318 K. In the meantime, the change in enthalpy (Δ*H*^0^) and the entropy(Δ*S*^0^) were calculated by eqs 7–9. In the following equations, *R* (8.314 J K^–1^ mol^–1^) is the molar gas constant; *T* (K) is adsorption temperature; and *K*_L_ (L mol^–1^) is the constant for the Langmuir equation.Gibbs free energy equations:
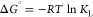
7
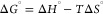
8According to eqs 7 and 8, we can conclude that
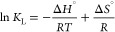
9

**Figure 7:**
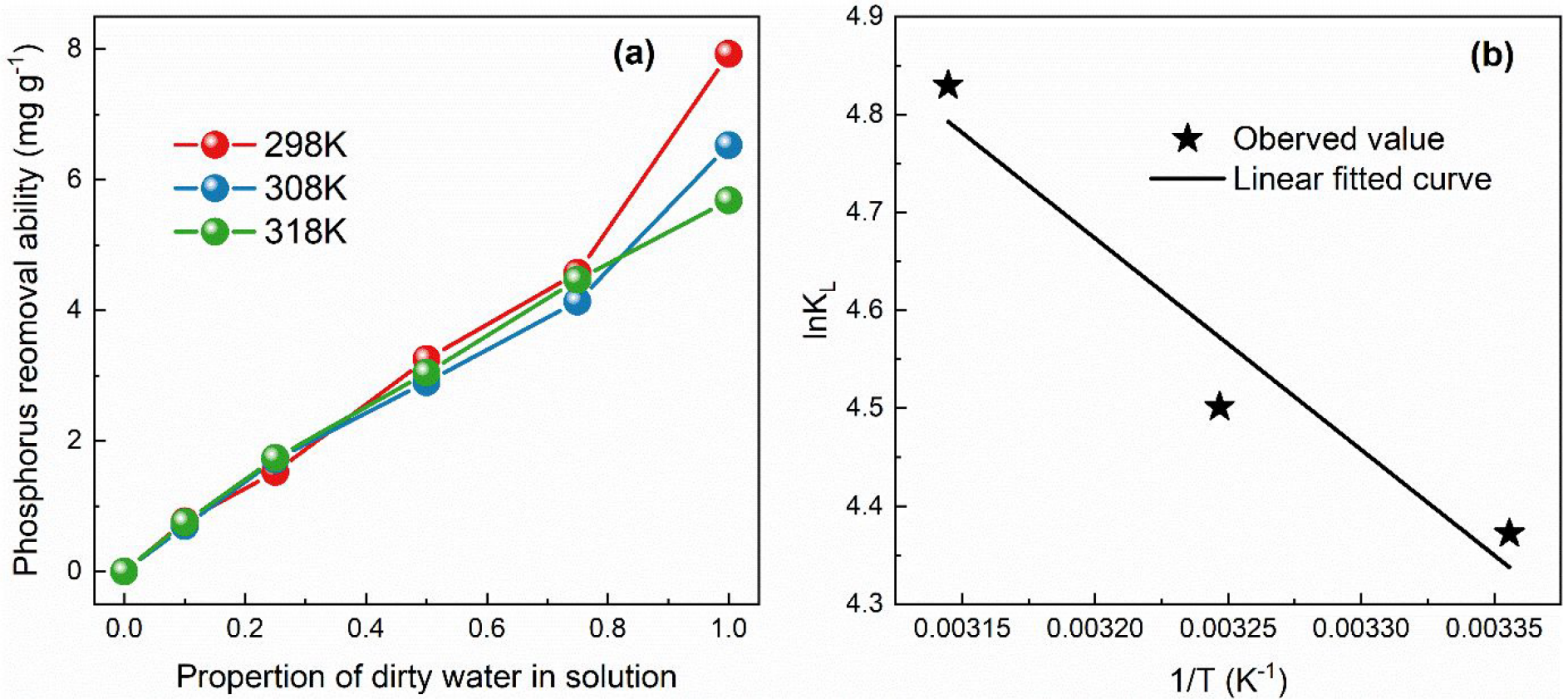
Adsorption thermodynamics of AD-Steam-1.0h phosphorus removal at different adsorption temperatures.

Table 4 shows the thermodynamic parameters for phosphorus removal by AD-Steam-1.0h. The Langmuir equation illustrates the isotherms of phosphorus adsorption by AD-Steam-1.0h under different temperature conditions. The Langmuir constant (*K*_L_) increases when the adsorption temperature changes from 298 K to 318 K. Besides, the absolute value of Gibbs free energy is also enhanced due to the variation of *K*_L_. As shown in Figure 7b, the curve of the Van ’t Hoff equation (eq 9) is plotted with three coordinate points to obtain the slope (−Δ*H*°/*R*) and intercept (Δ*S*°/*R*) of the curve of the first order function; then Δ*H*° and Δ*S*° are calculated. According to the results, Δ*H*° and Δ*S*° show values of 17.93 kJ mol^–1^ and 96.24 J mol^–1^ K^–1^, respectively. The phosphorus removal process by biochar is a spontaneous endothermic reaction because of the negative Gibbs free energy exhibition.^42,49^ Therefore, phosphorus removal by biochar is feasible from the thermodynamic point of view.

**Table 4:** Thermodynamic Parameters for Phosphorus Removal by AD-Steam-1.0h

temperature (K)	*K*_L_(L mol^–1^)	Δ*G*(kJ mol^–1^)	Δ*H*^0^(kJ mol^–1^)	Δ*S*(J mol^–1^ K^–1^)
298	79.21	–10.83	17.93	96.24
308	90.13	–11.53		
318	125.17	–12.77		

## 4 Conclusion

This work illustrates a promising anaerobic digestate-derived biochar sample for agricultural dirty water phosphorus removal. The physical-gas-activated biochar samples performed better than the chemical-activated samples from the aspect of phosphorus removal efficiency. AD-N_2_ shows the highest phosphorus removal ability of 8.99 mg g^–1^, and AD-Steam-1.0h also suggests an impressive phosphorus removal ability of 7.98 mg g^–1^. Moreover, kinetic studies prove that the removal of phosphorus from dirty water adsorption by AD biochar prefers the Elovich kinetic model because of the rough adsorbent surface. Furthermore, the phosphorus removal process by biochar is spontaneous and endothermic, according to the thermodynamic analysis. Therefore, using solid waste from the same institution to treat agricultural irrigation dirty water is a sustainable development concept, which may achieve substantial results in economy and efficiency

## Supporting Information Available

Additional methods details (phosphorus determination reagent and calibration curve determination), phosphorus calibration curve and linear fitting, SEM-EDX elemental mapping images, and average yearly concentration of components in the irrigation dirty water (PDF)
