# Supplementary material for: Phosphorus Removal from Dirty Farmyard Water by Activated Anaerobic-Digestion-Derived Biochar
Source: Ind Eng Chem Res. 2022 Dec 5;62(45):19216–24. doi: 10.1021/acs.iecr.2c02668 (PMC10655106; doi:10.1021/acs.iecr.2c02668)
Supplement: Supplementary file 1 — ie2c02668_si_001.pdf [file ie2c02668_si_001.pdf]

Supporting Information

Phosphorus removal from farmyard dirty water by activated anaerobic digestion biochar

Chen Zhang[a], Shuzhuang Sun[a], Shaojun Xu[b,c], Chris Johnston[d], Chunfei Wu[a]*

[a]*School of Chemistry and Chemical Engineering, Queens University Belfast, Belfast, UK BT7 1NN*

[b]*School of Chemistry, Cardiff University, Cardiff, CF10 3AT, UK*

[c]*UK Catalysis Hub, Research Complex at Harwell, Didcot, OX11 0FA, UK*

[d]*The Agri-Food and Biosciences Institute, Northern Ireland, BT9 5PX, UK*

*Corresponding authors: *Dr Chunfei Wu (E-mail: c.wu@qub.ac.uk)*

Phosphorus determination reagent

The phosphorus determination reagent is a chemical compound to characterize the contents of phosphate in solution. The fresh reagent is yellow-green colour; it gradually changes to dark yellow as time goes on.

Preparation of Phosphorus Determination Reagent: Mix the reagents in the following order and volume ratio: Distilled water: 3 mol L^{-1} H_2SO_4: 2.5% ammonium molybdate: 10% vitamin C = 2: 1: 1: 1

3 mol L^{-1} H_2SO_4 solution: 166mL of concentrated H_2SO_4 with a density of 1.84 g mL^{-1} is measured in a graduated cylinder and gradually added to a beaker containing 500mL of distilled water while stirring. After the solution is cooled, transfer it to a 1000mL volumetric flask. Add water to the mark and shake well.

2.5% ammonium molybdate: Weigh 2.5g of chemically pure ammonium molybdate, first add a small amount of distilled water in a small beaker to dissolve it, then transfer it to a 100mL volumetric flask, add distilled water to the mark, and shake to serve.

10% Vitamin C: Weigh out Vitamin C 1g, add 10mL of distilled water to dissolve, the solution must be prepared temporarily, and stored in a brown bottle, and can be stored in the refrigerator for one month.

Calibration curve determination

Weigh accurately analytically pure KH_2PO_4 0.4398g, dissolve in a small amount of distilled water, make up to volume with a 1000 mL volumetric flask, and shake to form a stock solution containing phosphorus 100 mg L^{-1}. Before using the simulated sewage, transfer 10 mL of the original solution into a 100 mL graduated cylinder with distilled water injecting a standard solution containing phosphorus 10 mg L^{-1}, named P10 simulated sewage.

After diluting simulated phosphorus sewage and preparing the solutions into six different sample vials to determine the calibration curve at a specific proportion by distilled water, simulated sewage, and determination reagent, a phosphorus determination was prepared would be shown in the support information section.

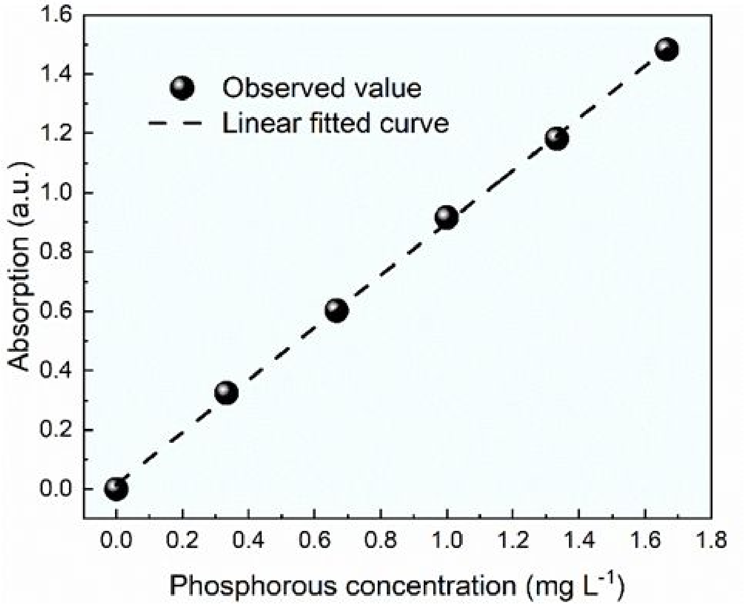

Figure S1: Phosphorus calibration curve and linear fitting.

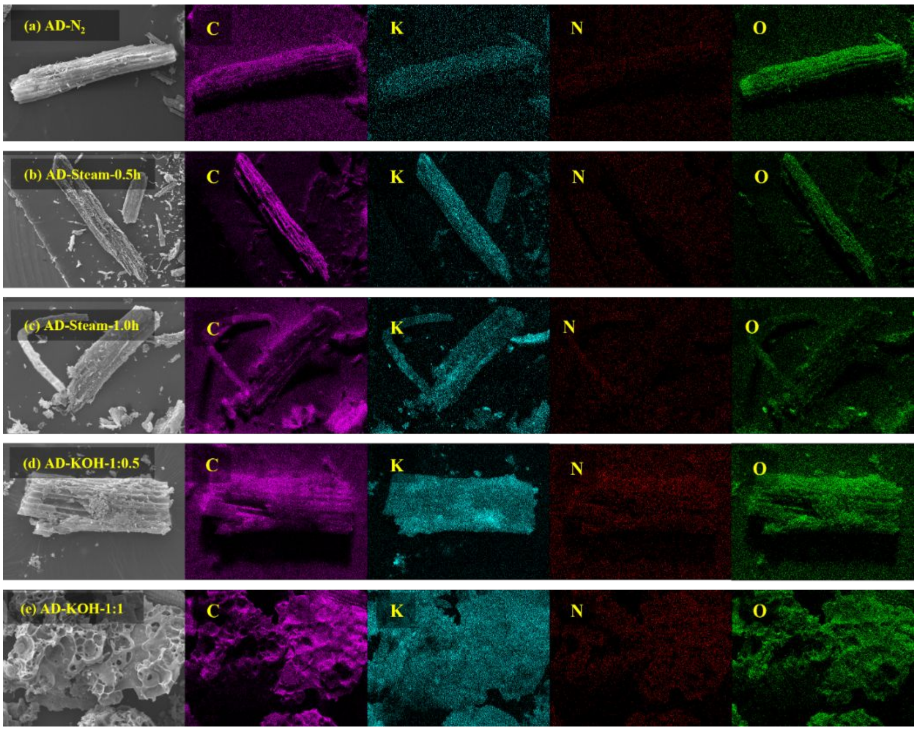

Figure S2: SEM-EDX elemental mapping images of C, K, N and O elements of (a) AD-N_2, (b) AD-Steam-0.5h, (c) AD-Steam-1.0h, (d) AD-KOH-1:0.5, and (e) AD-KOH-1:1.

Table S1: The average yearly concentration of components in the irrigation dirty water

Year	pH	BODs (mg L^{-1})	N (mg L^{-1})	P (mg L^{-1})	K (mg L^{-1})
2008	5.83	2935	172.5	67.3	327.5
2009	5.80	2336	102.7	42.4	292.3
2010	5.71	2720	170.1	61.8	531.6
2011	6.41	2785	197.5	50.5	604.8
P-value	0.472	0.031	0.107	0.489	0.107
Se	0.397	104.7	29.9	8.84	88.9
Lsd	1.33	333.5	95.7	28.27	284.5

P-value: significance at 5% probability level ($p<0.05$); Se: standard error; Lsd: least significant difference
